# An uncommon inheritance pattern in Niemann-Pick disease type C: identification of probable paternal germline mosaicism in a Mexican family

**DOI:** 10.1186/s12883-016-0649-5

**Published:** 2016-08-22

**Authors:** Marivi Cervera-Gaviria, Miguel Angel Alcántara-Ortigoza, Ariadna González-del Angel, Paola Moyers-Pérez, Blanca Gabriela Lizet Legorreta-Ramírez, Nancy Barrera-Carmona, Jaime Cervera-Gaviria

**Affiliations:** 1 Departamento de Genética Médica, Centro de Rehabilitación e Inclusión Infantil Teletón, Vía Gustavo Baz No. 219, Colonia San Pedro Barrientos, Tlalnepantla, Estado de México 54960 México; 2 Laboratorio de Biología Molecular, Departamento de Genética Humana, Instituto Nacional de Pediatría, Ciudad de México, México; 3 DNA-GEN, S.C. Centro de Alta Especialidad en Genética Humana, Ciudad de México, México; 4 Departamento de Rehabilitación Pediátrica, Centro de Rehabilitación e Inclusión Infantil Teletón, Estado de México, México; 5 Departamento de Neuropediatría, Centro de Rehabilitación e Inclusión Infantil Teletón, Estado de México, México; 6 Servicio de Medicina Interna, Sociedad de Beneficencia Española, Ciudad de México, México

**Keywords:** Germline mosaicism, Genetic counseling, Lysosomal storage disease, Niemann-Pick disease type C, *NPC1* mutations

## Abstract

**Background:**

Niemann-Pick disease type C (NP-C) is a fatal lysosomal neurodegenerative and neurovisceral disease. It is caused by defects in intracellular lipid trafficking, which lead to the accumulation of lipids and glycosphingolipids within the endosomes and lysosomes of affected individuals. Pathogenic variants of the *NPC1* or *NPC2* genes yield highly variable phenotypes with a time course that ranges from fetal onset (i.e., *hydrops fetalis*) to progressive dementia in adults. NP-C is typically inherited in an autosomal-recessive manner. To our knowledge, no previous report has identified germline mosaicism as an inheritance mechanism in NP-C.

**Case presentation:**

We report the case of a male Mexican patient with “variant” filipin staining and a juvenile form of NP-C attributed to compound heterozygosity for two previously reported pathogenic variants of *NPC1*: c.[1042C>T];[2780C>T] or p.[Arg348*];[Ala927Val]. The proband’s mother and healthy sister were heterozygous carriers of the c.2780C > T (exon 18) and c.1042C > T (exon 8) variants, respectively. However, direct sequencing of exons 8 and 18 of *NPC1* revealed no mutation in genomic DNA obtained from the father’s peripheral blood. DNA profiling ruled out the possibility of non-paternity. We were unable to obtain a sperm sample to demonstrate paternal gonadal mosaicism. *NPC1* haplotype analysis using 20 linked single nucleotide variants failed to yield sufficient information to document a p.(Arg348*) *NPC1* pathogenic variant-associated haplotype in the family.

**Conclusions:**

We propose that this case of NP-C involves paternal germline mosaicism. To the best of our knowledge, this has not previously been reported in NP-C.

## Background

Niemann-Pick disease type C (NP-C, MIM#257220) is a fatal neurovisceral lipid lysosomal storage disorder characterized by progressive neurological deterioration and premature death [1, 2]. The incidence of NP-C in European populations is about 1 in 120,000 newborns [1]. The disease is usually considered to be pan-ethnic, but its incidence is unknown in the Mexican population [1, 2]. NP-C reflects an inability to process cellular cholesterol: a defect in intracellular lipid trafficking leads to the accumulation of cholesterol and glycosphingolipids within the endosomal compartments and lysosomes of affected individuals [1–4].

NP-C is caused by mutations in the *NPC1* (95 %) and *NPC2* genes (5 %), and is classically inherited with an autosomal-recessive pattern [1]. The *NPC1* gene (18q11.2) spans 55.1 kb, includes 25 exons, and encodes the 13-transmembrane-domain NPC1 protein, which comprises 1,278 amino acids and is primarily localized to late endosomes [1, 3]. To date, nearly 60 polymorphisms and more than 300 disease-causing mutations have been identified in *NPC1*; of the latter, most (~70 %) are missense pathogenic variants that affect the cysteine-rich luminal domain and lead to variable clinical presentations [1, 2, 5].

The large number of compound heterozygous patients has made it difficult for researchers to determine any genotype-phenotype correlation for NP-C. However, genetic testing of each newly diagnosed patient and his/her first-degree relatives is highly advisable. The obtained information can be used to establish specific recurrence risks, identify carriers, offer prenatal diagnosis, and exclude uncommon inheritance mechanisms, such as the potential germline mosaicism identified in the present family.

## Case presentation

**Fig. 1:**
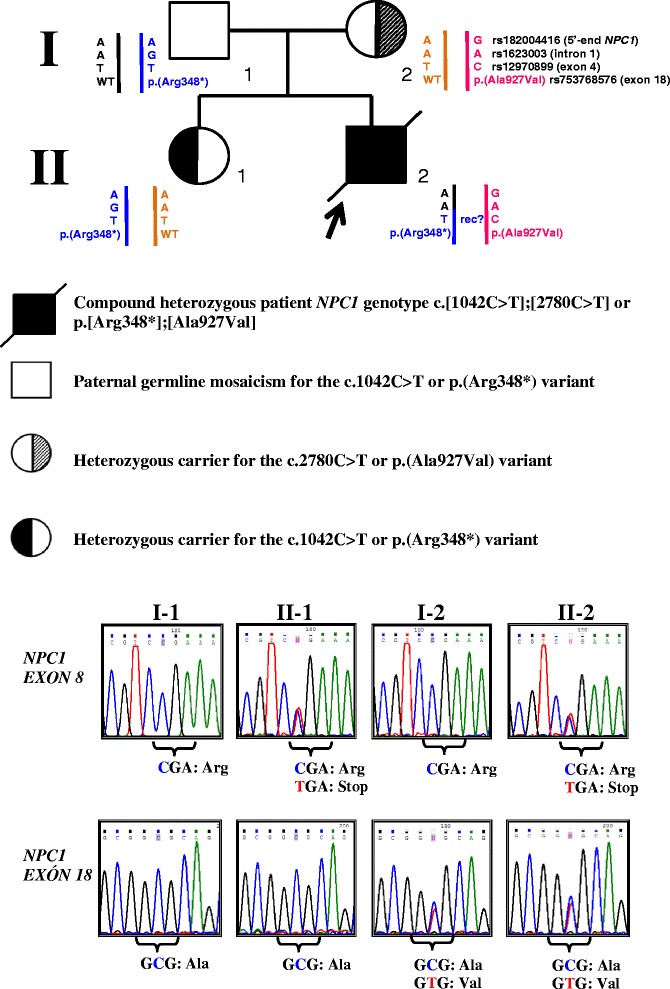
Pedigree of the described NP-C case, partial electropherograms (forward strands) of exons 8 and 18 of *NPC1*, and haplotype analysis of family members using the three informative intragenic markers. Sanger automated DNA sequencing of the entire coding sequence of *NPC1* in the proband (II-2) revealed the heterozygous compound genotype, c.[1042C > T];[2780C > T] or p.[Arg348*];[Ala927Val] (reference *NPC1* sequence: NM_000271.4). This result was confirmed using a second genomic DNA blood sample from II-2. To determine allelic segregation, we directly examined both pathogenic variants in leukocyte-derived genomic DNA obtained from the proband’s parents (I-1 and I-2) and healthy sibling (II-1). Obligate carrier status was confirmed in I-2, who was heterozygous for the *NPC1* c.2780C > T or p.(Ala927Val) (exon 18) variant, and II-1 was found to be an obligate carrier for the *NPC1* c.1042C > T or p.(Arg348*) (exon 8) variant. We assume the latter variant is of paternal origin, but it was not detected by automated bidirectional sequencing of total blood leukocyte-derived genomic DNA from I-1. Paternity testing through DNA profiling clearly confirmed the paternity of both siblings (data not shown). Our haplotype analysis failed to identify a common paternal haplotype for this mutation, although it did suggest the presence of a paternal recombination event (rec?). However, our molecular findings are consistent with the presence of paternal germline mosaicism for the c.1042C > T or p.(Arg348*) pathogenic variant. WT: wild-type *NPC1* allele

The proband was a 19-year-old boy of Mexican ancestry born from healthy non-consanguineous parents (Fig. 1). His clinical manifestations and analytic results are presented in Table 1, along with those previously reported in patients harboring at least one *NPC1* gene mutation in common with our patient [5–8]. His symptomatology initiated at 9 years old with progressive clumsiness; over the next six years, he developed cataplexy, progressive dysarthria, ataxia, fatigue and problems swallowing liquids. He had to leave school because of learning difficulties, memory loss, and impaired socialization. A clinical suspicion of NP-C at 18 years of age prompted a filipin staining test that yielded a “variant” phenotype [4]. Further molecular analyses confirmed the diagnosis of NP-C by exhibiting compound heterozygosity of *NPC1*: c.[1042C > T];[2780C > T] or p.[Arg348*];[Ala927Val] (Fig. 1). These pathogenic variants are located in exons 8 and 18, respectively, of the *NPC1* gene (Genbank reference sequence: NM_000271.4).

**Table 1:** Comparison of the clinical characteristics and genotypes of previously reported patients who share at least one mutation with the present case

	Present case	Meiner et al. 2001 [5]	Xiong et al. 2012 [6]	Jahnova et al. 2014 [7]			Piña et al. 2014 [8]
Case	1	2	3	4	5	6	7
Age at onset	9 years	20 years	8.5 years	14 years (sibling of cases 5 and 6)	27 years (sibling of cases 4 and 6)	20 years (sibling of cases 4 and 5)	24 years
Gender	Male	Male	Male	Male	Female	Female	Female
Family consanguinity	-	+	-	-	-	-	-
Progressive clumsiness	+	-	+	-	-	-	+
VSGP	+	+	-	-	-	-	+
Dysarthria	+	+	+	+	-	-	-
Cataplexy	+	-	-	-	-	-	-
Ataxia	+	+	+	-	-	-	+
Splenomegaly	+	+	-	+	+	+	+
Cerebellar atrophy	-	NR	+	NR	NR	NR	+
Behavioral anomalies	+	-	-	+	+	+	+
Depression	-	-	-	-	+	-	+
Schizophrenia	-	-	-	-	-	+	+
Bone marrow aspiration	Blue histiocytes	Blue histiocytes	Blue histiocytes	NR	Blue histiocytes	NR	Foamy histiocytes
NP-C index suspicion score [11] at diagnosis	142 points	NR	NR	NR	NR	NR	227 points
Filipin staining	“Variant”^a^	Positive	Not performed	“Variant”^a^	“Variant”^a^	“Variant”^a^	Positive
*NPC1* genotype	c.[1042C > T];[2780C > T] or p.[Arg348*];[Ala927Val]	c.[2780C > T];[2780C > T] or p.[Ala927Val];[Ala927Val]	c.[2777C > T];[2780C > T] or p.[Ala926Val];[Ala927Val]	c.[2780C > T];[2780C > T] or p.[Ala927Val];[Ala927Val]	c.[2780C > T];[2780C > T] or p.[Ala927Val];[Ala927Val]	c.[2780C > T];[2780C > T] or p.[Ala927Val];[Ala927Val]	c.[1042C > T];[3493G > A] or p.[Arg348*];[Val1165Met]
Family *NPC1* studies	Mother and healthy sister: obligate p.(Ala927Val) and p.(Arg348*) carriers, respectively Father: germline mosaicism for c.1042C > T (p.Arg348*) variant	NR	NR	NR	NR	NR	NR

^a^The “variant” filipin fibroblast profile of NP-C refers to situations in which supplementation with pure low-density lipoproteins yields a less intense and non-uniform pattern of perinuclear fluorescent vesicles in cells. This phenotype makes it impossible to establish an accurate percentage of NP-C “positive” cells. Moreover, the presence of these “variant” phenotype cells within total human serum causes the filipin staining pattern to overlap even further with the normal to very mildly abnormal perinuclear fluorescent vesicle distribution characteristic of NP-C [4]

Abbreviations: *NR* not reported, *VSGP* vertical supranuclear gaze paralysis, *(+)* present, and *(-)* absent

To provide the family with complete genetic counseling, we performed direct automated sequencing of exons 8 and 18 of the *NPC1* gene in genomic DNA obtained from blood leukocytes of the proband’s parents and healthy older sister (Fig. 1). The mother was found to be a heterozygous carrier of the c.2780C > T or p.(Ala927Val) *NPC1* allele located in exon 18. The healthy sister was found to be a heterozygous carrier of the c.1042C > T or p.(Arg348*) pathogenic variant in exon 8. Surprisingly, the father’s sample was not found to have any mutation in these exons. Paternity testing performed using 16 short tandem repeat markers (13 belonging to the CODIS system) confirmed the paternity of both siblings (data not shown).

To test whether the c.1042C > T or p.(Arg348*) variant shared a common *NPC1* haplotype, we subjected 20 closely linked or intragenic *NPC1* single nucleotide variants (tel-rs1620047, rs1652354, rs182004416, rs2981422, rs2303880, rs1623003, rs1788781, rs1367084, rs12970899, rs143656971, rs1805081, rs1652344, rs1788799, rs1805082, rs1140458, rs6507720, rs367911777, rs55724504, rs2510344, rs1805084-cen) to direct automated sequencing in all family members. Unfortunately, only the genotypes of markers rs182004416 (NM_000271.4:c.-1002A > G, 5′ end of *NPC1*), rs1623003 (NM_000271.4:c.57 + 1088G > A, intron 1) and rs12970899 (NM_000271.4:c.387T > C, exon 4) provided sufficient information to document phases in the family, and the analysis failed to identify a common haplotype for this pathogenic variant. Our results suggest the presence of a recombination event between markers rs1623003 and rs12970899 in the proband or his healthy carrier sister. Alternatively (though far less likely), the p.(Arg348*) variant could reside in different haplotypes of these two individuals (Fig. 1).

The patient died at 21 years of age due to respiratory distress secondary to bronchopneumonia. At the time of death, he scored 22 points on a modified disability scale for NP-C [9]. He had not received specific substrate-reduction therapy (Zavesca®, Miglustat) because the family could not afford the treatment.

## Conclusions

NP-C exhibits marked clinical variability and often remains undetected for many years, with an average delay in diagnosis of 5–6 years from the onset of neurological symptoms [10]. Consistent with this, our patient first exhibited symptomatology at 9 years of age but was not diagnosed with a clinical suspicion of NP-C for another 9 years. He exhibited the classical clinical manifestations of the juvenile form of the disease when he was examined at 18 years of age. His index suspicion score at diagnosis was 142 points (>70 points is highly suspicious for NP-C) [11].

Our molecular study of the patient’s first-degree relatives showed that the c.1042C > T or p.(Arg348*) and c.2780C > T or p.(Ala927Val) pathogenic variants resided in different *NPC1* alleles, confirming the compound heterozygous status diagnostic for NP-C. The c.2780C > T or p.(Ala927Val) is a missense transition-type pathogenic variant that affects the third endoluminal cysteine-rich domain of the protein, where most of the NP-C-causing mutations have been described [1, 2, 5]. A homozygous c.2780C > T or p.(Ala927Val) NP-C-affected male patient of Arab Muslim ancestry was previously reported [5]. This individual presented a clinical picture similar to that of the present case (Table 1), including the presence of mild splenomegaly and ataxia. Another report described a family with three cases of adult NP-C, all of whom were homozygous for c.2780C > T [7]; similar to the present case, these patients showed “variant” filipin staining, splenomegaly, and cognitive impairment. Based on the present and prior observations, we conclude that the juvenile/adult form of NP-C associated with the c.2780C > T mutation usually manifests with a “variant” filipin staining phenotype (Table 1).

The c.1042C > T or p.(Arg348*) mutation is a nonsense transition-type variation that was previously reported as a pathogenic variant [8, 12]. Interestingly, this mutation was also reported in a female Mexican patient who had a confirmed diagnosis of NP-C, a juvenile presentation, and a clinical picture very similar to that described herein [8]. Unlike the present case, however, no molecular study was performed to confirm that both parents were obligate carriers [8].

NP-C was previously thought to show strictly classical autosomal-recessive inheritance, with both parents being obligate carriers of the disease. Our current results suggest the presence of paternal germline mosaicism. Unfortunately, the father was no longer available for us to confirm somatic and/or gonadal mosaicism by studying additional tissues (e.g., sperm cells) or employing deep resequencing or ultra-sensitive methodologies (i.e., quantitative real-time PCR) to estimate the proportion of the mutated allele [13]. It should be noted that the term “germline mosaicism” should not be taken as implying that the pathogenic variation is confined to gonadal tissue; indeed, it may also appear in somatic tissues other than the original sample source [13, 14].

We analyzed intragenic markers in an effort to document a common haplotype for the p.(Arg348*) variant, as this would presumably support its origin from a single paternal germ cell line. However, the 10 intragenic markers located closest to the p.(Arg348*) variant (lying between exons 5 and 25) failed to yield informative genotypes, rendering us unable to integrate the phases of *NPC1* or determine the presence/absence of a variant-linked haplotype with any certainty. Three markers located toward the 5′ end of the *NPC1* gene provided sufficient information to suggest that there was a recombination event in the paternal germline (Fig. 1). An alternative (but less likely) possibility is that the p.(Arg348*) variant may reside in two distinct paternal *NPC1* haplotypes.

In recent years, mixed somatic and germline mosaicism has been recognized as an important and relatively common mechanism through which genetic disorders originate [13–15]. Such mosaicism has been reported in diseases with autosomal-recessive inheritance, including Gaucher disease (MIM#230800) [16] and Alport syndrome (MIM#203780) [17]. In most cases, these mutations occur via mechanisms such as unequal homologous crossing-over, excision of intrachromatid loops, inadequate incorporation of nucleotides during the replication process, or deamination of 5-methylcytosine [15]. In the case of Gaucher disease, two families were recently described [16]; each had a single affected patient, and in both cases only the paternal mutated allele was identified in leukocyte-derived DNA. No mutation was detected in blood-derived DNA samples from the mother, sister, or maternal grandmother of either proband. Maternity was confirmed with a 99.7 % certainty in both cases, prompting the authors to postulate that the presence of Gaucher disease in the patients was due to maternal germline mosaicism or (less likely) a *de novo* p.Leu444Pro mutation in the maternal germlines of both families [16]. These possibilities were not confirmed with further molecular testing, but should be considered in the context of genetic counseling. In the current case, based on the presence of the c.1042C > T variant in both the patient and his healthy sister (but not their father), we conclude that the mutated allele was most likely to have been inherited through paternal germline mosaicism (Fig. 1). It seems far less probable that there were two *de novo* mutations in the paternal germline.

In conclusion, our finding of probable germline mosaicism in NP-C has major clinical implications for genetic counseling. Currently, parental DNA is not evaluated in all cases, as exemplified by the fact that the carrier status of both parents was not examined in the four reported families that share at least one of the pathogenic variants identified in our patient (Table 1). Our novel finding supports the contention that in NP-C, clinicians must confirm the carrier status of both parents and analyze other first-degree relatives in order to provide families with accurate genetic counseling regarding their recurrence risk in future pregnancies.

## Abbreviations

(-), absent; (+), present; NP-C, Niemann-Pick disease type C; NR, not reported; rec?, probably reflects a recombination event; VSGP, vertical supranuclear gaze paralysis
